# Phosphate regulator PhoP directly and indirectly controls transcription of the erythromycin biosynthesis genes in *Saccharopolyspora erythraea*

**DOI:** 10.1186/s12934-019-1258-y

**Published:** 2019-11-27

**Authors:** Ya Xu, Di You, Li-li Yao, Xiaohe Chu, Bang-Ce Ye

**Affiliations:** 1 Institute of Engineering Biology and Health, Collaborative Innovation Center of Yangtze River Delta Region Green Pharmaceuticals, College of Pharmaceutical Sciences, Zhejiang University of Technology, Hangzhou, 310014 Zhejiang China; 2 Lab of Biosystems and Microanalysis, State Key Laboratory of Bioreactor Engineering, East China University of Science and Technology, Shanghai, 200237 China

**Keywords:** Erythromycin biosynthesis, Antibiotics, Phosphate metabolism, PhoP regulator, Transcriptional regulation

## Abstract

**Background:**

The choice of phosphate/nitrogen source and their concentrations have been shown to have great influences on antibiotic production. However, the underlying mechanisms responsible for this remain poorly understood.

**Results:**

We show that nutrient-sensing regulator PhoP (phosphate regulator) binds to and upregulates most of genes (*ery* cluster genes) involved in erythromycin biosynthesis in *Saccharopolyspora erythraea*, resulting in increase of erythromycin yield. Furthermore, it was found that PhoP also directly interacted with the promoter region of *bldD* gene encoding an activator of erythromycin biosynthesis, and induced its transcription. Phosphate limitation and overexpression of *phoP* increased the transcript levels of *ery* genes to enhance the erythromycin production. The results are further supported by observation that an over-producing strain of *S. erythraea* expressed more PhoP than a wild-type strain. On the other hand, nitrogen signal exerts the regulatory effect on the erythromycin biosynthesis through GlnR negatively regulating the transcription of *phoP* gene.

**Conclusions:**

These findings provide evidence that PhoP mediates the interplay between phosphate/nitrogen metabolism and secondary metabolism by integrating phosphate/nitrogen signals to modulate the erythromycin biosynthesis. Our study reveals a molecular mechanism underlying antibiotic production, and suggests new possibilities for designing metabolic engineering and fermentation optimization strategies for increasing antibiotics yield.

## Background

In actinomycetes, nitrogen and phosphate control of the biosynthesis of antibiotics, pigments, immunomodulators, and many other types of secondary metabolites is a well-known phenomenon [8, 13, 18, 21]. Regulatory network based on nitrogen regulator GlnR and phosphate regulator PhoP as well as the underlying mechanisms of how nutritional signals exert the regulatory effect on the biosynthesis of secondary metabolites have widely been investigated in *Streptomyces*. The *Streptomyces coelicolor* A3(2) *ΔglnR* mutant strain was clearly affected with respect to antibiotic production. No production of the pigmented antibiotics actinorhodin and undecylprodigiosin was observed on solid medium and in liquid culture. Complementation with the *glnR* gene encoding nitrogen regulator restored the wild-type phenotype [17]. Moreover, a similar effect on antibiotic production was also reported for the GlnR protein of the rifamycin producer *Amycolatopsis mediterranei*. The deletion of the *A. mediterranei glnR* gene resulted in a reduced rifamycin production. The complementation of a *S. coelicolor glnR* mutant strain FS10 with the *glnR* gene of *A. mediterranei* led to an excessive production of undecylprodigiosin, while actinorhodin production was blocked [24]. Apparently, GlnR influences antibiotic production of *A. mediterranei* and *S. coelicolor* A3(2). It was indicated that GlnR is a global regulator with a dual-functional impact on nitrogen metabolism and related antibiotic production. However, it is unclear how the GlnR-mediated regulation is connected to antibiotic production. GlnR may be important for the induction of a general stress response, triggered by nutrient limitation, which finally activates antibiotic biosynthesis [24]. Recently, microarray analysis and chromatin immuno-precipitation (ChIP) identified thirty-six putative GlnR target genes with GlnR binding sites throughout the *Streptomyces venezuelae* genome. GlnR binds to the intergenic region between the divergently transcribed *jadR1* and *jadR2* genes, which encode transcriptional regulators that activate and repress, respectively, expression of the jadomycin biosynthetic genes [11].

The phosphate-sensing PhoR–PhoP system is also involved in regulating the production of actinorhodin in *Streptomyces lividans* [15] and undecylprodigiosin in *S. coelicolor* [14]. The biosynthesis of the antifungal polyene macrolide pimaricin in *Streptomyces natalensis* is very sensitive to phosphate concentration in the culture broth. Concentrations of inorganic phosphate as low as 2 mM drastically reduced pimaricin production. No transcripts for all the pimaricin biosynthesis (*pim*) genes including the pathway-specific positive regulator *pimR* could be detected at 10 mM phosphate. A PhoP-deleted mutant reveals increased pimaricin yield and is less sensitive to phosphate concentration. No putative PhoP-binding sequences were found in the promoter regions of any of the *pim* genes, suggesting that phosphate control of these genes is mediated indirectly by PhoR–PhoP [10]. Martin et al. have found that PhoP regulatory effect on antibiotic biosynthesis may be exerted through signaling cascades of PhoP-AfsS-AfsR-SARP (Streptomyces antibiotic regulatory proteins, such as ActII-orf4 and RedD) in *Streptomyces* [9]. The studies also observed that the expression of *glnR* gene and some other GlnR-regulated genes is repressed by PhoP in *S. coelicolor* [4, 9]. These findings reveal crosstalk between global regulators (PhoP, GlnR, and AfsR) in *S. coelicolor* that controls the expression of genes associated with secondary metabolite biosynthesis. However, no phosphate-related gene was found in the GlnR regulon, suggesting that GlnR has no direct effect on phosphate metabolism and demonstrating that the crosstalk between GlnR and PhoP is not reciprocal [16]. Interestingly, more recently, we found that GlnR negatively regulates the transcription of *phoP* gene in *Saccharopolyspora erythraea*. There appears to be reciprocal regulatory crosstalk between GlnR and PhoP in *S. erythraea*, unlike *S. coelicolor* and *S. lividans* [23].

The choice of nitrogen/phosphate source and their concentrations have a great influence on the erythromycin production in *S. erythraea*. Reeve et al. investigated the effect of glucose, nitrogen, and phosphorus sources on the timing and extent of erythromycin production [12]. High-phosphate cultures (10–100 mM) repressed erythromycin biosynthesis and the transcription of *ery* genes. The production of erythromycin and transcription of *ery* cluster genes were induced in low-phosphate cultures (< 1 mM). In the same study, the data demonstrated that NH_4_NO_3_ and other ammonium salts gave a considerable lag before growth started, and cultures grown on it produced no or low levels of erythromycin. No *ery* transcript could be detected in the ammonium grown culture. This conclusion was also supported by results of recent experiments, in which the erythromycin production was strongly inhibited by ammonium [1, 5]: These results suggest that ammonium and phosphate impact the transcription of *ery* cluster genes and that nitrogen/phosphate metabolism and biosynthesis of erythromycin are deeply interconnected. These observations provide evidence that *S. erythraea* may possess a molecular mechanism involving crosstalk between nitrogen/phosphate metabolism and erythromycin biosynthesis. However, the homologous gene for *afsS* was not found in the *S. erythraea* genome and no SARP was identified as being responsible for erythromycin biosynthesis. The underlying mechanisms of how nutritional signals exert the regulatory effect on the biosynthesis of erythromycin remain poorly understood.

In this study, we identified four putative PhoP-boxes (binding sites) in the promoter regions of *sace_0712* encoding erythromycin esterase and operon *eryBVI*-*BVII,* as well as in the intergenic regions between the operons *eryAI*-*G* and *eryBIV*-*BVII*, *eryBI* and *eryBIII*-*F* involved in erythromycin biosynthesis. It was found that the phosphate-sensing PhoP, as an activator, strongly regulated the transcription of these genes (19 genes of all 22 genes in *ery* cluster) responding to phosphate availability. Moreover, PhoP directly also regulated the transcription of *bldD*, whose product was a key regulator of actinomycetes development and activated all genes of *ery* cluster [2]. Nitrogen signal exerts the regulatory effect on the transcription of *ery* cluster through GlnR negatively regulating the transcription of *phoP* gene. The results suggested that PhoP played an important role in the control of biosynthesis of erythromycin in response to environmental phosphate/nitrogen signals. Our findings reveal a novel molecular mechanism underlying the antibiotics production, and suggests new possibilities for designing metabolic engineering and fermentation optimization strategies for increasing antibiotics yield.

## Results

### Effect of phosphate on erythromycin production

**Fig. 1:**
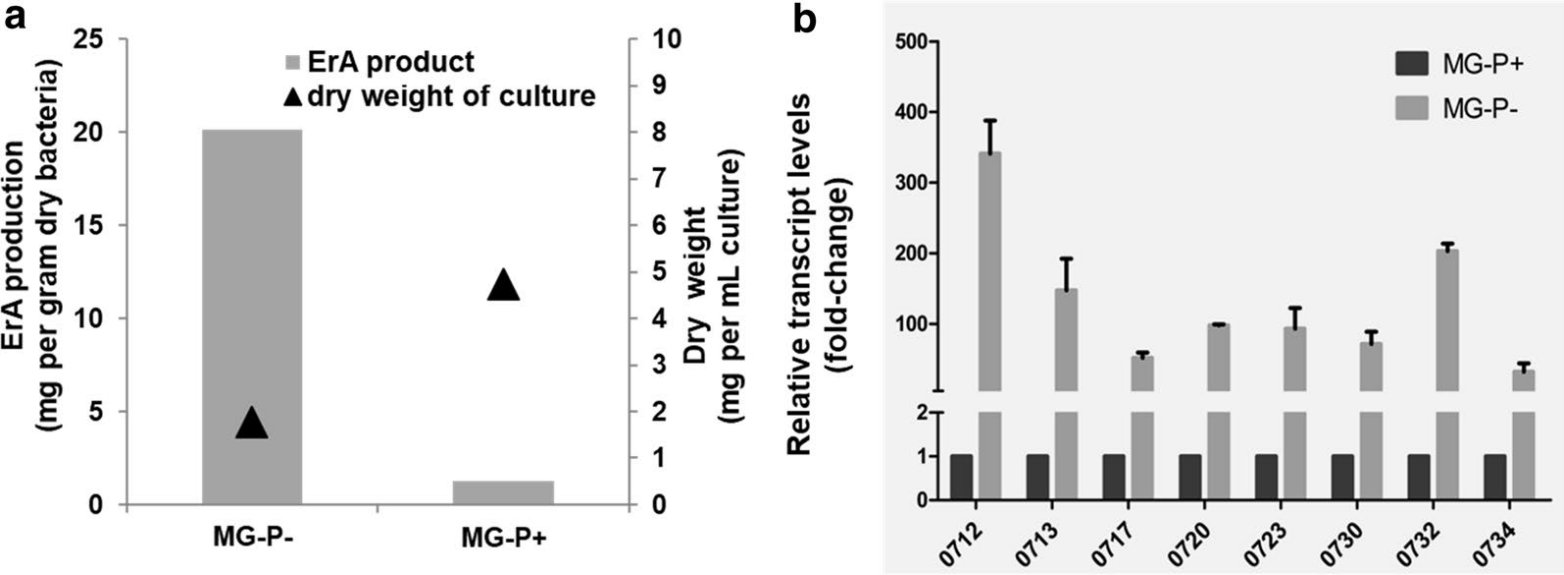
Effects of phosphate concentration on erythromycin (ErA) production and *ery* transcription. **a** ErA production and biomass yield of *S. erythraea* strain in low (40 μM (MG-P−) and high (10 mM, MG-P+) concentrations of phosphate in MG medium (50 g/L starch, 60 mM glutamate). **b** The transcriptional profiles of some genes in *ery* cluster responding to high (MG-P+) and low (MG-P−) phosphate

In *S. erythraea*, the erythromycin production and the levels of *ery* mRNAs were regulated by nitrogen/phosphate sources and their concentrations [12]. Recently, we found that phosphate regulator PhoP exerted positive control on phosphate metabolism and erythromycin biosynthesis [23]. To further investigate the effect of phosphate concentrations on biosynthesis of erythromycin, the *S. erythraea* wild-type strain NRRL2338 was grown in high-phosphate MG medium (MG-P+, 60 g/L starch, 60 mM glutamate, and 10 mM phosphate) and low-phosphate MG medium (MG-P−, 60 g/L starch, 60 mM glutamate, and 50 μM phosphate) for 3 days. Production of erythromycin (ErA) and accumulation of biomass were determined. As shown in Fig. 1a, production of erythromycin in low-phosphate MG medium is approximately 20-fold higher than that in high-phosphate MG medium. The high phosphate greatly repressed the biosynthesis of erythromycin. The transcription levels of genes in *ery* cluster in response to phosphate availability were examined. As expected, these genes were strongly induced during low-phosphate condition (Fig. 1b). The quantitative RT-PCR experiments indicated that transcript levels of all the transcript units (TUs) in the *ery* cluster significantly increased by above 100-fold. These results further demonstrated that phosphate limitation activated erythromycin production, thereby indicating the importance of phosphate source sensing/utilization in *S. erythraea* antibiotic production.

### Identification of four binding sites of PhoP in the promoter regions of ery cluster

**Fig. 2:**
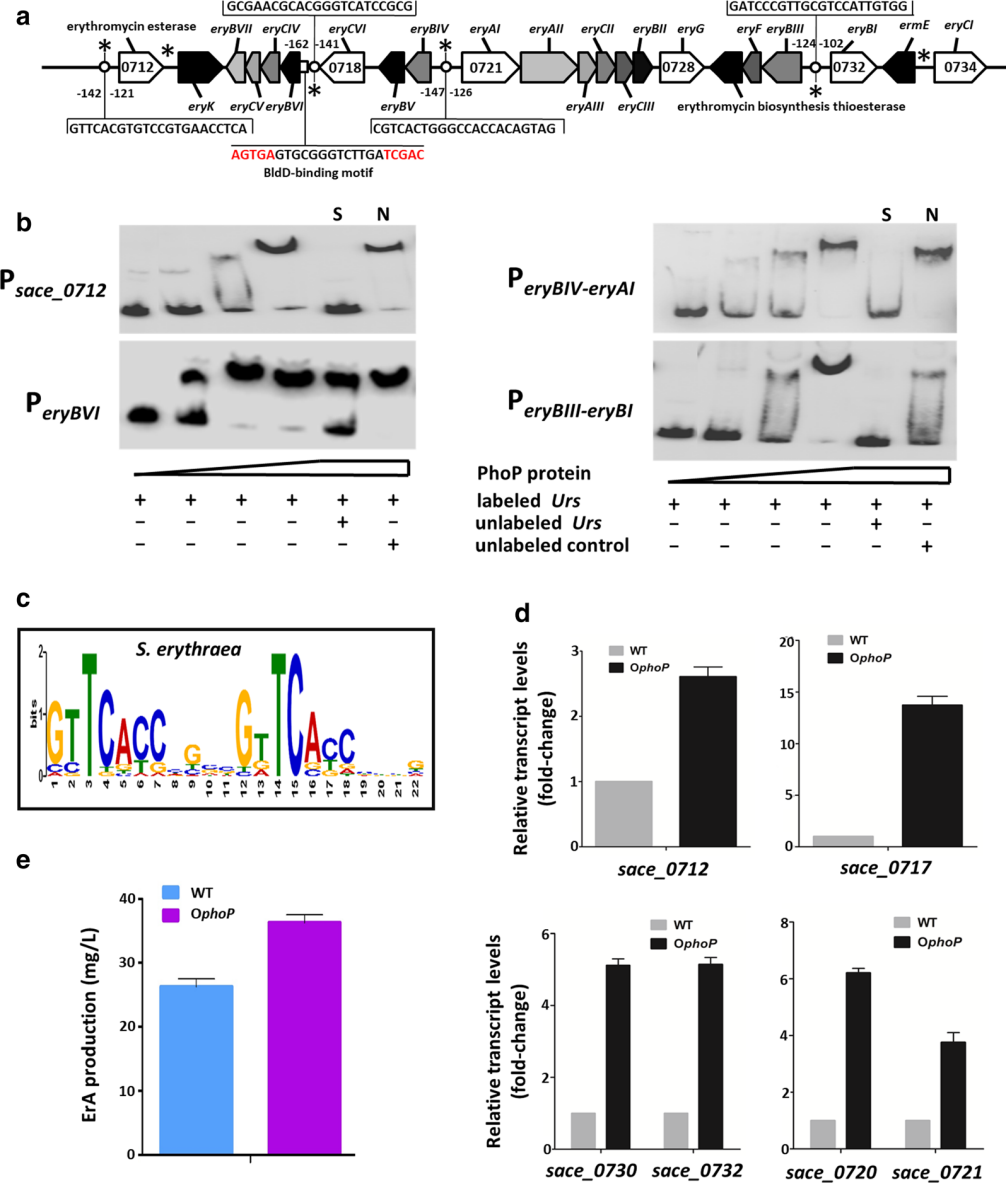
PhoP directly binds to promoter regions in *ery* genes cluster. **a** The cis-element analysis about the erythromycin biosynthetic gene cluster (*ery*). **b** EMSAs of His_6_-PhoP protein of *S. erythraea* with regulatory regions of *sace_0712, eryBVI, eryBIV*-*eryAI,* and *eryBIII*-*eryBI*; unlabeled specific probe (100-fold) (S) or nonspecific competitor DNA (100-fold, salmon sperm DNA) (N) was added. The concentrations of His_6_-PhoP protein in each lane from left to right was 0 µM, 0.2 µM, 0.4 µM, 0.8 µM, 0.8 µM, 0.8 µM. **c** The PHO box in *S. erythraea.* The height of each letter is proportional to the frequency of the base, and the height of the letter stack is the conservation in bits at that position. **d** The transcriptional profiles of *ery* genes in WT and PhoP-overexpressed strain (O*phoP*). The value for the transcription level in O*phoP* was a relative value compared to WT. **e** The ErA production of WT and O*phoP* strains in TSB medium for 3 days

The organization of *ery* cluster was shown in Fig. 2a. There are six regulatory regions (marked with asterisk in Fig. 2a) in the *ery* cluster, including the promoter regions of *sace_0712* gene, *eryK* gene, and operon *eryBVI*-*BVII,* as well as in the intergenic regions of *eryBIV*-*eryAI*, *eryBIII*-*eryBI,* and *ermE*-*eryCI*. In order to determine whether the PhoP protein binds directly to these regulatory regions of *ery* cluster, gradient EMSA assays were performed using purified His-PhoP protein. To verify the specificity of the binding, unlabeled specific probe (100-fold) or nonspecific competitor DNA (100-fold, salmon sperm DNA) was used. Obvious shift bands were observed in EMSA assays for *sace_0712*, *eryBVI*, *eryBIV*-*eryAI,* and *eryBIII*-*eryBI* following addition of His-PhoP (Fig. 2b). In *S. erythraea*, PhoP-binding motif (PHO-box) was identified [23]. The PHO-box is formed by two conserved direct repeats units (DRUs) of 11-nucleotides, with each one formed by an identical seven-nucleotide sequence (GtTCacc), followed by a four-nucleotide less-conserved tail region, and bound by a PhoP protein (Fig. 2c). Using MEME/MAST tools (http://meme.sdsc.edu) and the PREDetector software program, four putative PhoP-boxes were found in the regulatory regions of genes, including *sace_0712* (two DRUs: GTTCACGTGTC CGTGAACCTCA), *eryBVI* (two DRUs: GCGAACGCACG GGTCATCCGCG), *eryBIV*-*eryAI* (two DRUs: CGTCACTGGGC CACCACAGTAG), and *eryBIII*-*eryBI* (two DRUs: GATCCCGTTGC GTCCATTGTGG) (Fig. 2a). The regulatory regions with the putative PhoP-boxes controlled six transcript units (Tus), *sace_0712* gene, *eryBVI* operon (4 genes), *eryBIV* operon (3 genes), *eryAI* operon (7 genes), *eryBIII* operon (3 genes), *eryBI* gene. To further examine the regulatory effect of PhoP on transcription of *ery* genes in *S. erythraea*, we tried to construct *phoP*-deletion mutant and *phoP* overexpressed strain (O*phoP*). Regretfully, our attempts to delete *phoP* gene were unsuccessful. The O*phoP* and wild-type (WT) strains were grown at 30 °C in TSB media for 48 h. The overexpression of *phoP* resulted in a 2.5- to 14-fold (2.5-fold for *sace_0712*, 14-fold for *eryBVI*, fivefold for *eryBIV*, fivefold for *eryAI*, 6.2-fold for *eryBI,* and 3.7-fold for *eryF* of *eryBIII* operon) increase in induction of *ery* genes (Fig. 2d). These results demonstrated that PhoP exhibits a regulatory function as a transcriptional activator of *ery* cluster genes involved in biosynthesis of erythromycin by directly upregulating the expression of six TUs.

To investigate the effect of PhoP on erythromycin production, the amount of erythromycin A (ErA) were determined in the O*phoP* and wild-type (WT) strains grown at 30 °C in TSB media for 3 days. We found that production of erythromycin in the O*phoP* strain increased by approximately 40% compared to the wild type strain (Fig. 2e).

### The regulatory effect of nitrogen on erythromycin production is mediated by PhoP

**Fig. 3:**
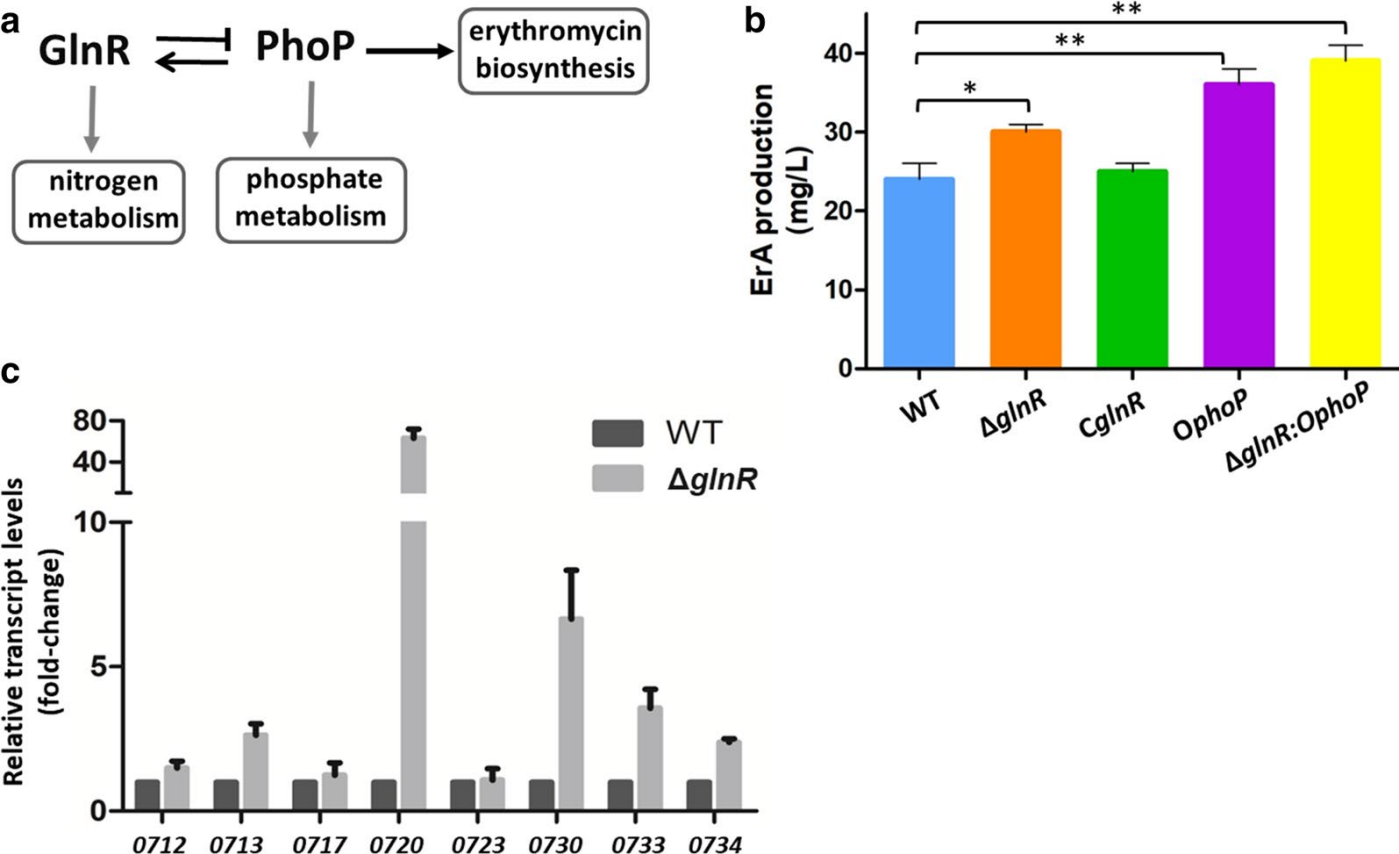
The regulatory effect of nitrogen on erythromycin production is mediated by PhoP. **a** GlnR–PhoP-ery cascaded regulation; **b** The ErA production of WT, Δ*glnR*, C*glnR*, O*phoP*, and Δ*glnR:*O*phoP* cultivated in TSB medium for 3 days. **c** The transcriptional profiles of *ery* cluster genes in WT and Δ*glnR* cultivated in TSB medium for 3 days. *P < 0.05, **P < 0.01

Nitrogen source and GlnR have a regulatory effect on the erythromycin production in *S. erythraea* [22]. However, it remains unclear how the GlnR-mediated nitrogen metabolism is connected to biosynthesis of erythromycin. No GlnR-binding motif was observed in the regulatory regions of *ery* cluster, indicating the effect of GlnR on erythromycin production was indirect. Our recent research demonstrated that *S. erythraea* revealed the reciprocal crosstalk between PhoP and GlnR: PhoP activated *glnR* transcription, whereas GlnR directly repressed *phoP* transcription [23], suggesting that the regulatory effect of nitrogen on erythromycin production may be mediated by PhoP (GlnR–PhoP-ery cascaded regulation) (Fig. 3a). As shown in Fig. 3b, when *glnR* was deleted, the titer of ErA increased to nearly 31 mg/L, nearly 23% higher than the value obtained in WT strain. The titer of ErA reversely reduced to the similar level with WT strain when the *glnR* was complemented to the null mutant (C*glnR*). ErA production of *phoP*-overexpressed Δ*glnR* (Δ*glnR:*O*phoP*) was further improved by 10% compared to the *phoP*-overexpressed strain (O*phoP*). To investigate the regulatory effects of GlnR on *ery* genes transcription in *S. erythraea*, the *glnR*-deleted mutant (Δ*glnR*) and wild type strain (WT) were cultivated in TSB media, cells harvested for RNA extraction at 72 h, and *ery* transcription analyzed using RT-PCR. Increases in transcription of most *ery* genes were observed in the Δ*glnR* mutant strain, indicating that *ery* cluster was repressed by GlnR (Fig. 3c).

### PhoP indirectly controls erythromycin biosynthesis by regulating the transcription of *bldD*

**Fig. 4:**
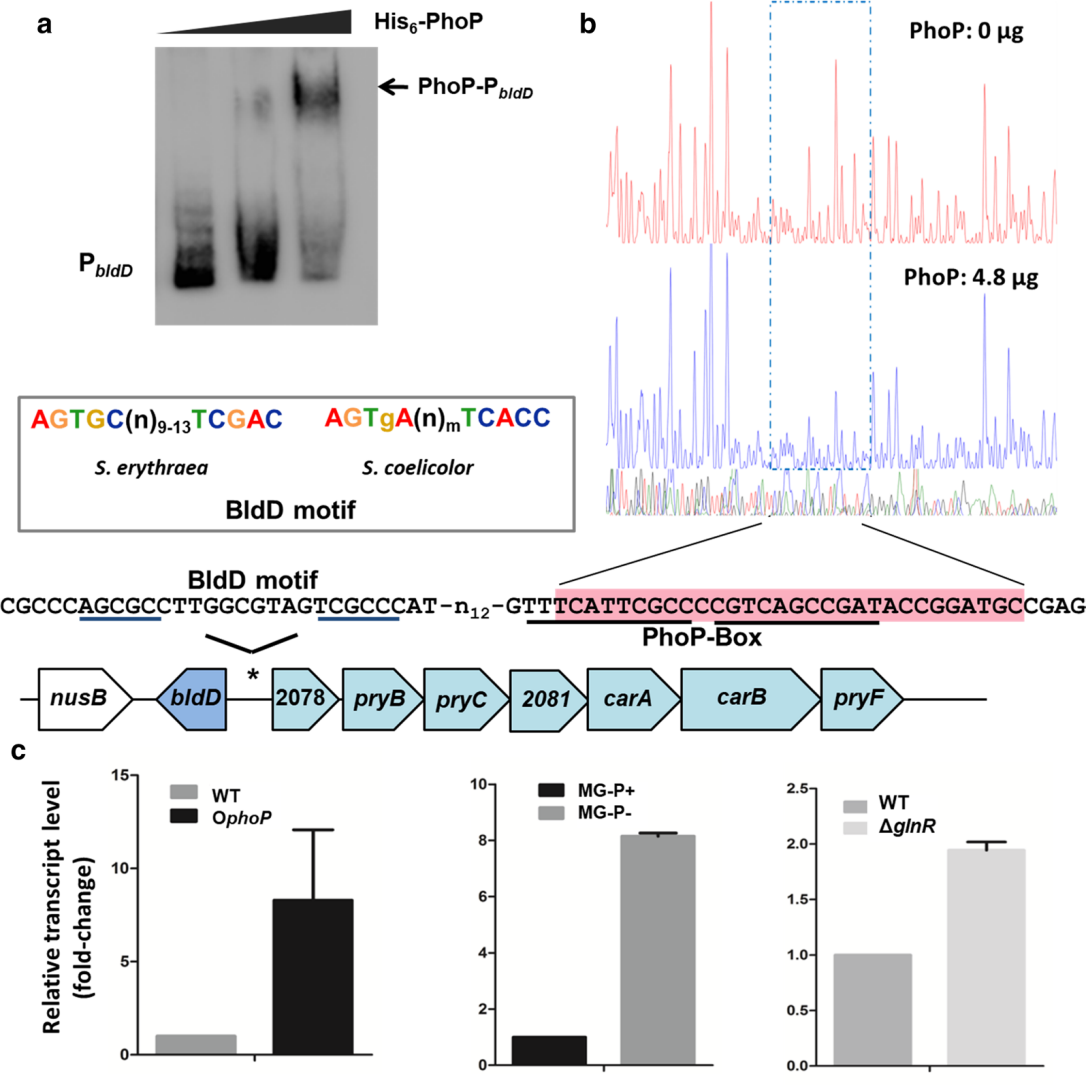
PhoP regulates the transcription of *bldD* in *S. erythraea*. **a** Electrophoretic mobility shift assays of His-PhoP to promoter region of *bldD* by incubating the bio-labeled DNA with 1 µM protein and a 200-fold excess of nonspecific competitor DNA (sperm DNA). P_*bldD*_ represented bio-labeled DNA sequence located in region from − 300 to + 50 of *bldD* gene. **b** The footprinting assay for promoter region of *bldD* with PhoP, and the pink solid rectangle refers to region protected by PhoP. Genetic organization of *bldD* and regulatory sequence were shown. The underline referred to the potential binding sites of BldD and PhoP. The consensus sequences of BldD binding motif in *S. erythraea* and *S. coelicolor* were shown inside the gray frame. **c** The transcriptional profiles of *bldD* gene in WT, *glnR*-deleted mutant (Δ*glnR*) and PhoP-overexpressed strain (O*phoP*). The transcriptional responses of *bldD* gene to high (MG-P+) and low (MG-P−) phosphate was also shown

BldD, a key developmental regulator encoded by sace_2077, was found to be able to bind all the five main promoter regions in the *ery* cluster and promote erythromycin biosynthesis [2]. Interestingly, we found that PhoP bind directly to the promoter regions of *bldD* gene (Fig. 4a). To identify the exact DNA sequences that were protected by PhoP in the promoter region of *bldD*, a DNase I footprinting assay using purified recombinant His-PhoP and a fluorescent FAM-labeled probe was performed. With the addition of His-PhoP (4.8 μg), a clearly protected region of about 30 nt was detected (Fig. 4b). Within the protected region, the putative PhoP-box consisting of two PhoP-binding motif (**TTTCATTCGCC**C**CGTCAGCCGAT**) was observed. In *S. erythraea* the expression of *bldD* is controlled by BldD itself [2]. The putative BldD-binding site (**AGCGC**-n_10_-**TCGCC**) was identified in upstream of *bldD* gene; which was highly similar to the consensus AGTGC(n)_9_TCGAC and AGTgA(n)_m_TCACC (m = 0–16) deduced in *S. erythraea* and *S. coelicolor* [3]. BldD-binding site is located in close proximity to the PhoP-box (separated by 15 bp) (Fig. 4b). RT-PCR experiments showed that the overexpression of PhoP resulted in the induction of *bldD* (eightfold), indicating that PhoP activated *bldD* transcription (Fig. 4c). The transcriptional response of *bldD* gene in wild-type strain was investigated under high (10 mM, MG-P+) and low (40 μM, MG-P−) phosphate in MG medium. The *bldD* transcript level was significantly increased (about eightfold) in phosphate-limited medium (Fig. 4c). In addition, the Δ*glnR* strain also revealed twofold increase on transcription level of *bldD*. These observations suggested that PhoP indirectly controlled erythromycin biosynthesis by regulating the transcription of *bldD*.

### More PhoP protein was observed in high-yield *S. erythraea* strain

**Fig. 5:**
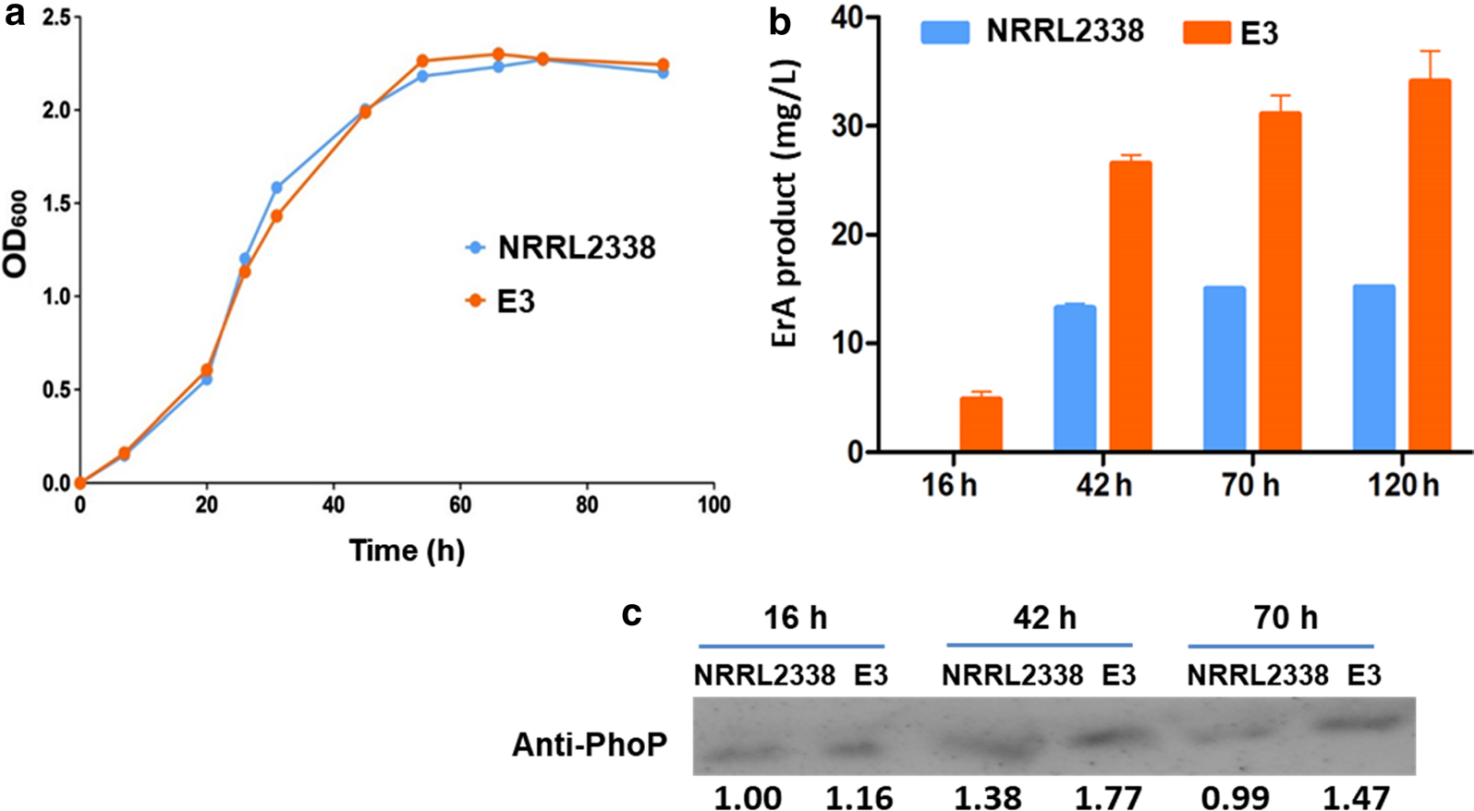
High-yield *S. erythraea* strain contains higher abundance of PhoP protein. **a** The growth curves of high-yield strain E3 and low-yield strain NRRL2338 in TSB. **b** Titers of erythromycin of two strains in TSB. **c** Western blot of lysates (8 μg of total protein) of the E3 and NRRL2338 strains at 16 h, 42 h, and 70 h with polyclonal antibody for PhoP. The band intensities were quantified by densitometry using ImageJ software

Finally, we examined the amount of PhoP protein in high-yield (HY) and low-yield (LY) strains of *S. erythraea*. An industrial *S. erythraea* strain E3 which has been widely used for erythromycin fermentation was employed as HY strain, while *S. erythraea* NRRL2338 as LY strain. Cultures of the LY and HY strains in a liquid, rich medium (TSB) were sampled at 16, 42, 70, and 120 h. The growth curves observed for HY and LY strains in TSB medium are presented in Fig. 5a. No change of growth in two strains was observed. Strain E3 produced erythromycin earlier (at 16 h) than strain NRRL2338, and yielded more erythromycin at each time point (Fig. 5b). We investigated the expression of PhoP in the HY and LY strains by using Western blots. Polyclonal anti-PhoP antibody detected PhoP in three lysates at 16 h, 42 h and 70 h which represented late lag phase, exponential growth phase, and stationary phase, respectively. The results demonstrated that high-yield strain contained higher abundance of PhoP than low-yield strain (Fig. 5c).

## Discussion

### PhoP plays a key role in cross-talk of phosphate metabolism and erythromycin biosynthesis in *S. erythraea*

**Fig. 6:**
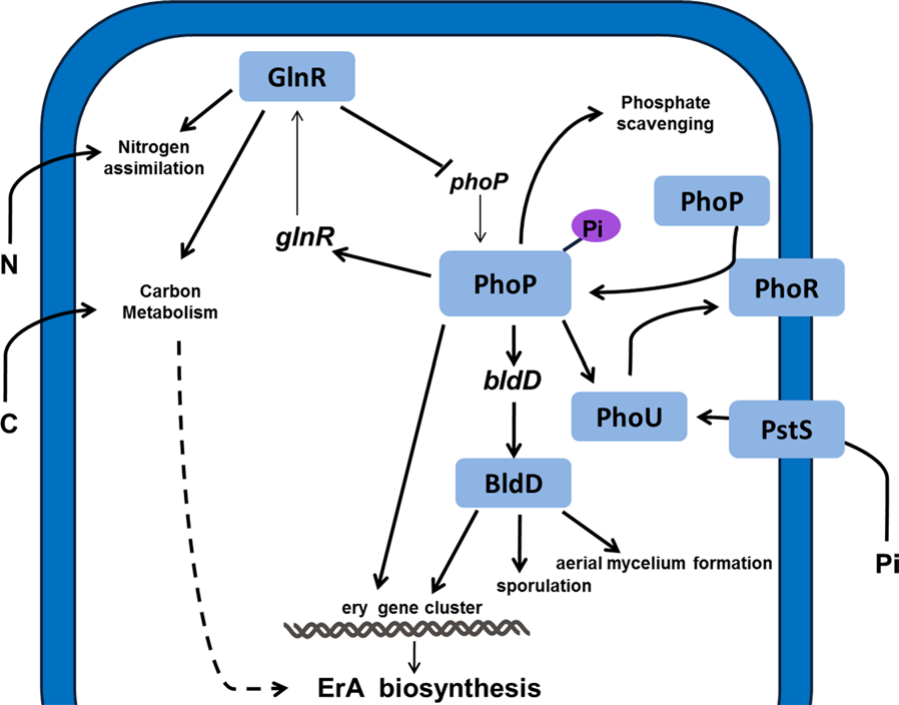
The PhoP-mediated cross-talk of primary metabolism and erythromycin biosynthesis in *S. erythraea*. Thick arrows refer to positive regulation; the thin arrows refer to transcription; the lines ended by a perpendicular short line refer to negative regulation

In the past decade, Martin group has proposed that PhoP plays a central role in primary and secondary metabolism in *S. coelicolor*. A complete nutrient-sensing signal transduction pathway, PhoP-AfsR-AfsS-SARP, was elucidated to demonstrate that the phosphate regulatory effect on secondary metabolism was exerted through interaction PhoP with AfsR and AfsS regulators [9]. However, the homologous gene for *afsS* was not observed in the *S. erythraea* genom*e* and no pathway-specific SARP regulator was identified as being responsible for erythromycin biosynthesis. So far the signaling system of PhoP-AfsR-AfsS-*ery*, controlling expression of genes involved in secondary metabolism has not been found. In the present study, we demonstrated that the phosphate-sensing PhoP, as an activator, strongly and directly regulated six transcript units involved in erythromycin biosynthesis, including *sace_0712* gene, *eryBVI* operon (4 genes), *eryBIV* operon (3 genes), *eryAI* operon (7 genes), *eryBIII* operon (3 genes), and *eryBI* gene. Most of *ery* cluster (19 genes of all 22 genes in *ery* cluster) were subject to regulation by PhoP, indicating that PhoP controlled biosynthetic cluster of erythromycin. Interestingly, PhoP directly also up-regulated the transcription of *bldD*, whose product was a key regulator of actinomycetes development and activated all genes of *ery* cluster [2]. Recent researches observed that overproducing strains showed lower expression level of *bldD* gene than wild-type strains, opposite to the *ery* genes [1, 5]. The regulatory effect of BldD on *ery* cluster remains still unclear. Phosphate limitation and overexpression of *phoP* increased the transcript levels of *ery* genes to enhance the erythromycin production. These observations demonstrated that PhoP exerted direct regulatory effect (PhoP-*ery*) or indirect regulatory effect mediated by BldD (PhoP-BldD-*ery*) on erythromycin biosynthesis. These findings revealed that PhoP played an important and central role in mediating the interplay between phosphate metabolism and secondary metabolism in *S. erythraea* by integrating phosphate signals to modulate the erythromycin biosynthesis (Fig. 6).

On the other hand, BldD is a developmental transcription factor [2], and plays important roles in many diverse processes, sporulation, aerial mycelium formation, antibiotic production, and extracellular matrix in *actinomycetes*. Our data showed that PhoP directly activates expression of BldD, indicating that PhoP has an extensive function on many facets of the biological process, such as morphological differentiation and development, beyond the primary and secondary metabolisms.

### PhoP mediates the regulatory effects of nutrients on erythromycin production in *S. erythraea*

The biosynthesis of erythromycin is also highly regulated by type and concentration of the nitrogen and carbon sources. However, not much is known on the actual mechanisms behind the observed effects on antibiotic formation, e.g. what the molecular mechanism is that underlies repression at high ammonia concentrations. In our previous studies we had identified phosphate regulator PhoP and nitrogen regulator GlnR in *S. erythraea,* and showed that PhoP and GlnR both collaboratively regulate the transcription of *glnR* and some nitrogen metabolism-related genes. Meanwhile, GlnR negatively controlled phosphate metabolism through its binding to the promoter of *phoP*-*phoR,* indicating that the two global regulators played reciprocal regulatory roles associated with nitrogen and phosphate metabolism in *S. erythraea* [22, 23]. More interestingly, GlnR directly regulated the transcription of three genes (*gltA*-*2*, *citA*, and *citA4*) which encode citrate synthase as the first enzyme of the TCA cycle. Acetyl-CoA is fed into the TCA cycle by citrate synthase, providing succinyl-CoA which can be converted to methymalonyl-CoA and propionyl-CoA as important precursors for erythromycin biosynthesis [7]. More recently, we found that GlnR regulates uptake and utilization of ABC-transported carbohydrates in actinomycetes, demonstrating that GlnR serves a role beyond nitrogen metabolism, and mediates critical functions in carbon metabolism and crosstalk of nitrogen- and carbon-metabolism pathways [6]. Taken together, nitrogen/carbon signals exert the regulatory effect on the erythromycin biosynthesis through GlnR negatively regulating the transcription of *phoP* gene (Fig. 6). It seems that the regulatory mechanism of erythromycin biosynthesis is much more complicated than expected, involving three global regulators (GlnR, PhoP, and BldD).

In conclusion, we found that phosphate-sensing regulator PhoP directly and indirectly activates the biosynthesis of erythromycin at the transcriptional level in *S. erythraea*. PhoP is well-positioned to link the nutritional status of the cell to the regulation of erythromycin production. These findings indicated a possible GlnR–PhoP–BldD-*ery* regulatory network in coordinating the primary nutrient metabolism and erythromycin biosynthesis responding to the nitrogen/phosphate sources availability, here provide a starting point for understanding the complex regulation of erythromycin biosynthesis. Our study reveals a molecular mechanism underlying the antibiotics production, and suggests new possibilities for designing new strategies for strain improvement and fermentation optimization strategies for increasing antibiotics yield.

## Methods

### Bacterial strains, plasmids, and growth conditions

The bacterial strains used in this study are listed in Table 1. The seeds of *S. erythraea* strains were grown on agar plates of the medium [10 g cornstarch, 10 g corn steep liquor, 3 g NaCl, 3 g (NH_4_)_2_SO_4_, 2 g CaCO_3_, and 20 g agar per liter of distilled H_2_O, pH 7.2] at 30 °C for sporulation. *E. coli* strains were grown at 37 °C in liquid or onto solid LB medium. All media were sterilized by autoclaving at 121 °C for 30 min. The EVANS and TSB media were described as the literatures [22, 23].

**Table 1:** Strains and plasmids used in this work

Strain/plasmids	Relevant characteristics	Source or reference
Strains		
*S. erythraea* NRRL2338	Used as parental strain, wild type	DSM 40517
*E. coli* DH5α	F^−^Ø80d lacZΔM (lacZYA -argF)U169 deoR	Invitrogen
*E. coli* BL21(DE3)-6965	The strain for expression of PhoP	This report
*E. coli* BL21(DE3)	F′ompTr-_B_m-_B_(λDE3)	This report
*S. erythraea OphoP*	The strain for over-expression of *phoP,* NRRL2338 integrated with pIB139-6965	This report
*S. erythraea* ∆*glnR*	NRRL2338 *glnR::tsr* (*glnR* null mutant)	This report
*S. erythraea* C*glnR*	*glnR complementary strain,* ∆*glnR* carrying pIB-*glnR*	This report
*S. erythraea* ∆*glnR:* *OphoP*	The strain for over-expression of *phoP*, ∆*glnR* integrated with pIB139-6965	This report
Plasmids		
pET28a	Expression vector	Novagen
P6965	pET28a derivative carrying *phoP* of *S. erythraea*	This report
pIB139	*Escherichia coli*–*S. erythraea* integrative shuttle vector containing a strong constitutive	[20]
pIB139-6965	pIB139 carrying an extra *phoP* for the gene overexpression	This report
pIB-*glnR*	pIB139 carrying an extra *glnR* for the gene overexpression	This report

### Overexpression and purification of PhoP protein

*Escherichia coli* cells transformed with p6965 plasmid, *E. coli* BL21 (DE3)-6965, were grown in LB medium at 37 °C in an orbital shaker (250 rpm.) to an OD_600_ of 0.6. The expression of *phoP* was induced by IPTG addition (0.1 mM final concentration) for 6–8 h. Cells were harvested by centrifugation and washed twice with PBS buffer (pH 8.0) and broken by ultrasonic cell crusher. Cell debris and membrane fractions were separated from the soluble fraction by centrifugation (45 min; 15,000 rpm; 4 °C). His-tagged PhoP (His_6_-PhoP) was purified by Ni–NTA Superflow columns (Qiagen, Germany). The protein presented a maximal peak of elution at around 250 mM imidazole (in 50 mM NaH_2_PO_4_, 300 mM NaCl, pH 8.0). Fractions containing His_6_-PhoP were pooled and dialyzed in Buffer D (50 mM Tris, 0.5 mM EDTA, 50 mM NaCl, 20% glycerol, 1 mM DTT, pH 8.0) at 4 °C and stored at − 80 °C. The purified His_6_-PhoP protein was assessed by sodium dodecyl sulfate polyacrylamide gel electrophoresis (SDS-PAGE). Protein concentration was determined with BCA Protein Assay Kit with BSA as the standard.

### Electrophoretic mobility shift assay (EMSA)

The putative promoter regions of the *ery* cluster genes and *bldD* gene (the upstream 350 bp from about − 300 to 50) were amplified by PCR using the primers listed in Additional file 1: Table S1. PCR products were labeled with biotin using a universal biotinylated primer (5′ biotin-AGCCAGTGGCGATAAG 3′). The biotin-labeled PCR products were identified by agarose gel electrophoresis and purified using a PCR Purification kit (Shanghai Generay Biotech Co., Ltd) as EMSA probes. The concentrations were determined with a microplate reader (Biotek, USA). EMSAs were carried out using a Chemiluminescent EMSA Kit (Beyotime Biotechnology, China), according to the manufacturer’s protocol. The binding reaction contained 10 mM Tris∙HCl pH 8.0, 25 mM MgCl_2_, 50 mM NaCl, 1 mM DTT, 1 mM EDTA, 0.01% Nonidet P40, 50 μg/mL poly[d(I-C)], 10% glycerol. Biotin-labeled DNA probes were incubated individually with varying amounts of PhoP protein at 25 °C for 20 min. For control groups, unlabeled specific probe (200-fold) or nonspecific competitor DNA (200-fold, sonicated salmon sperm DNA) was used. After binding, the samples are separated on a native PAGE gel in ice-bathed 0.5× Tris–borate-EDTA at 100 V and bands are detected by BeyoECL Plus. The amount of all DNA probes used in EMSA was about 1 pmol, and the protein’ amount was approximately 50 pmol.

### Computional analysis

The MEME/MAST tools (http://meme.sdsc.edu) and the PREDetector software program were used to search GlnR/PhoP binding motif binding sites in the upstream region of *ery* genes in *S. erythraea*. The operon prediction of *ery* cluster genes and *bldD* was performed with MicrobesOnline database (http://www.microbesonline.org/operons/).

### DNase I footprinting assay

The promoter region of *bldD* was PCR amplified with primers *P2077F and P2077R* (Additional file 1: Table S1), and the amplicon was cloned into the T-vector pUC18B-T (Shanghai Biotechnology Corporation, SBC). The obtained plasmids were used as templates for further preparation of biotin-labeled probes with universal primer primers bio-Tprimer (Additional file 1: Table S1). After agarose gel electrophoresis, the FAM-labeled probes were purified using a QIAquick Gel Extraction Kit (Qiagen) and quantified with a NanoDrop 2000C (Thermo, USA). For each assay, 200 ng of each probe was incubated with different amounts of His-PhoP in a total volume of 40 µL in EMSA buffer (Beyotime Biotechnology, China). After incubation for 30 min at 25 °C, 10 µL of solution containing about 0.015 units DNase I (Promega, Madison, WI, USA) and 100 nM freshly prepared CaCl_2_ was added, and the sample was further incubated for 1 min at 25 °C. The reaction was stopped by adding 140 µL DNase I stop solution (200 mM unbuffered sodium acetate, 30 mM EDTA, and 0.15% SDS). Samples were extracted with phenol/chloroform and precipitated with ethanol. The pellets were dissolved in 30 µL Milli-Q water. The preparation of the DNA ladder, electrophoresis, and data analysis were performed as previously described [19], except that the GeneScan-LIZ500 size standard (Applied Biosystems, Foster City, CA, USA) was used.

### RNA preparation and real-time RT-PCR

*Saccharopolyspora erythraea* was grown for 2 days at 30 °C in seed medium. Next, 0.5 mL of the preculture was used to inoculate the TSB medium or modified Evans medium (30 mL). Samples were collected at different time points. Cell pellets were collected after 20 min of centrifugation at 3000 rpm. Total RNA was prepared using RNeasy Mini Kit (Qiagen, Valencia, CA). The RNA integrality was analyzed by 1% agarose gel electrophoresis and the RNA concentration was determined by microplate reader (BioTek, USA). Total RNA (1 μg) extracted from liquid cultures was reverse transcribed using a PrimeScript™ RT Reagent Kit with gDNA Eraser (Takara, Shiga, Japan) for real-time RT-PCR, the DNase digestion was performed to remove genomic DNA before reverse transcription for 5 min at 42 °C. All procedures above are following the manufacturer’s instructions. PCR reactions were performed with primers listed in Additional file 1: Table S2. SYBR premix Ex Taq™ GC Kit (Perfect Real Time, Takara) was used for real-time RT-PCR, and about 100 ng cDNA was added in 20 μL volume of PCR reaction. The PCR was conducted using CFX96 Real-Time System (Bio-Rad, USA) and the PCR conditions were 95 °C for 5 min; then 40 cycles of 95 °C for 5 s, 60–64 °C for 30 s; and an extension at 72 °C for 10 min.

### Erythromycin determination by HPLC

The high-performance liquid chromatography (HPLC) condition was as follows: mobile phase [(50 mM K_2_HPO_4_; pH 8.0): acetonitrile 50:50], detection wavelength (210 nm, UV–VIS SPD-20A, Shimadzu), chromatographic column (5 μm Inertsil ODS-SP, 4.6 × 250 mm, Shimadzu), and rate (0.8 mL/min). The fermentation samples were prepared by lyophilization and the following methanol-dissolving.

### Western blot

The protein concentrations of the samples were determined using BCA Protein Assay Kit (TIANGEN) with BSA as the standard. Protein samples extracted by ultrasonication were separated by SDS–PAGE and then transferred to a PVDF membrane for 30 min at 100 V. The membrane was blocked at 24° C in 1× TBST (20 mM Tris–HCl, pH 7.5, 150 mM NaCl, and 0.1% Tween-20) containing 5% non-fat dry milk (NFDM) for 2 h. Then Anti-PhoP antibody diluted 1:15,000 in TBST/0.5%NFDM was used. After incubation at 4 °C for overnight, the blot was washed with TBST for 3 times. The membrane was incubated with horseradish peroxidase-conjugated anti-mouse IgG (1 μg/mL in TBST with 3% BSA) at ambient temperature for 2 h. The ECL system (CTB, USA) was used for signal detection according to the manufacturer in conjunction with a luminescent image analyzer, Bio-Imaging Systems (DNR Bio-Imaging Systems, ISRAEL).

## Supplementary information

**Additional file 1: Table S1.** The primers used for amplification of gene upstream regions in EMSAs. **Table S2.** The primers used in RT-qPCR.

## Data Availability

All data generated or analysed during this study are included in this published article.
