# Supplementary material for: Phosphate regulator PhoP directly and indirectly controls transcription of the erythromycin biosynthesis genes in Saccharopolyspora erythraea
Source: Microb Cell Fact. 2019 Nov 27;18:206. doi: 10.1186/s12934-019-1258-y (PMC6880422; doi:10.1186/s12934-019-1258-y)
Supplement: Supplementary file 1 — Additional file 1: Table S1. The primers used for amplification of gene upstream regions in EMSAs. Table S2. The primers used in RT-qPCR. [file 12934_2019_1258_MOESM1_ESM.docx]

**Additional Information**

**Phosphate regulator PhoP directly and indirectly controls transcription of the erythromycin biosynthesis genes in *Saccharopolyspora erythraea***

 Ya Xu^1,2#^, Di You^2#^, Li-li Yao^2^, Xiaohe Chu^1^*, Bang-Ce Ye^1,2^*

^1^Institute of Engineering Biology and Health, Collaborative Innovation Center of Yangtze River Delta Region Green Pharmaceuticals, College of Pharmaceutical Sciences, Zhejiang University of Technology, Hangzhou 310014, Zhejiang, China.

^2^Lab of Biosystems and Microanalysis, State Key Laboratory of Bioreactor Engineering, East China University of Science and Technology, Shanghai 200237, China.

**Table S1.** The primers used for amplication of gene upstream regions in EMSAs

| Primer Name | Sequence(5'-3') |
| --- | --- |
| ery-0712F | AGCCAGTGGCGATAAGCGAACACAAAGCACC |
| ery-0712R | AGCCAGTGGCGATAAGGTGCAGGTACTGCC |
| ery-0713F | AGCCAGTGGCGATAAGCGTTCGGGAGGTGGAT |
| ery-0713R | AGCCAGTGGCGATAAGAACTTCGTCGATGGTG |
| ery-0717F | AGCCAGTGGCGATAAGCACGTGGAGCACTACGA |
| ery-0717R | AGCCAGTGGCGATAAGTGCGGTCTGCGAGGAT |
| ery-0718F | AGCCAGTGGCGATAAGCAGTTGCACATGAATG |
| ery-0718R | AGCCAGTGGCGATAAGAACCCGCCCTCGTACAT |
| ery-0721F | AGCCAGTGGCGATAAGAAGCGCCCAGAAGGGTG |
| ery-0721R | AGCCAGTGGCGATAAGGACTGTCGGAGAGCTT |
| ery-0723F | AGCCAGTGGCGATAAGATCGGCCTCCCTACAG |
| ery-0723R | AGCCAGTGGCGATAAGCACCTTCTCGCTGTCAGT |
| ery-0728F | AGCCAGTGGCGATAAGATCGAGGCCTACGAGAAG |
| ery-0728R | AGCCAGTGGCGATAAGGTCCTGCAACGCTGA |
| ery-0732F | AGCCAGTGGCGATAAGCGATGCGGATGACCA |
| ery-0732R | AGCCAGTGGCGATAAGTCCGGGGGAACACTC |
| ery-0733F | AGCCAGTGGCGATAAGGCCCGTGCAGGTACCA |
| ery-0733R | AGCCAGTGGCGATAAGCTCGTCCGAACTGCTC |
| 2077F | AGCCAGTGGCGATAAGGGGTCCTCTCCCGACG |
| 2077R | AGCCAGTGGCGATAAGATAGCGCGGAGCTTGC |
| bio-Tprimer | biotin-AGCCAGTGGCGATAAG |

**Table S2.** The primers used in RT-qPCR

| Primer Name | Sequence(5'-3') |
| --- | --- |
| RT0712F | CTTCGCCGTTCGGGAGGTG |
| RT0712R | GGTCCAGGCCGGGAGCTTG |
| RT0713F | CCGATGGACCACGAGCAGTT |
| RT0713R | GCCAGGACAAGGCGGGAG |
| RT0717F | CCTGCTGGTCAAGGACATCG |
| RT0717R | CACATAGGCCGGTCGGAACT |
| RT0720F | GCCGCCCGTGTTGCTCTAC |
| RT0720R | CCCTCGTCGGTGGCTTTGC |
| RT0723F | CGCTGCCGACGTATCCGT |
| RT0723R | GCCTCGCACAGCATTTCCGT |
| RT0730F | TCCAGGACGACGATGACGG |
| RT0730R | GGGTGGGTGAGCAGCAGGTA |
| RT0732F | GGAGGGCATCTTCTCGGGCTAC |
| RT0732R | GTCGGTGCGCTCGAACGTG |
| RT0734F | CACTACCAGACGGAACTGAAGGAC |
| RT0734R | TTCTCGCAGCGGAGGACGA |
| RT2077F | AAGTCAGGCGGGCGGTG |
| RT2077R | CTTTGGTGGCGGGCTCG |
| RT7101F | GCAGGAGGTCTGGGGCTACG |
| RT7101R | GACGGGCGGACGAACTTGTAG |
| RT6965F | CGGTCAGGAGGCGTTGGAG |
| RT6965R | GCTGCTTGCAGACGTCGGT |
| RT8101F | GTTGCGATGCCGTGAGGT |
| RT8101R | CGGGTGTTACCGACTTTCA |
